# Increased usability, algorithmic improvements and incorporation of data mining for structure calculation of proteins with REDCRAFT software package

**DOI:** 10.1186/s12859-020-3522-x

**Published:** 2020-12-03

**Authors:** Casey Cole, Caleb Parks, Julian Rachele, Homayoun Valafar

**Affiliations:** Department of Computer Science and Engineering, University of South Carolina, M. Bert Storey Engineering and Innovation Center, 550 Assembly St, Columbia, SC 29201 USA

**Keywords:** Protein folding, Residual dipolar coupling (RDC), Residual dipolar coupling based residue assembly and filter tool (REDCRAFT), Secondary structure, Data mining

## Abstract

**Background:**

Traditional approaches to elucidation of protein structures by Nuclear Magnetic Resonance spectroscopy (NMR) rely on distance restraints also known as Nuclear Overhauser effects (NOEs). The use of NOEs as the primary source of structure determination by NMR spectroscopy is time consuming and expensive. Residual Dipolar Couplings (RDCs) have become an alternate approach for structure calculation by NMR spectroscopy. In previous works, the software package REDCRAFT has been presented as a means of harnessing the information containing in RDCs for structure calculation of proteins. However, to meet its full potential, several improvements to REDCRAFT must be made.

**Results:**

In this work, we present improvements to REDCRAFT that include increased usability, better interoperability, and a more robust core algorithm. We have demonstrated the impact of the improved core algorithm in the successful folding of the protein 1A1Z with as high as ±4 Hz of added error. The REDCRAFT computed structure from the highly corrupted data exhibited less than 1.0 Å with respect to the X-ray structure. We have also demonstrated the interoperability of REDCRAFT in a few instances including with PDBMine to reduce the amount of required data in successful folding of proteins to unprecedented levels. Here we have demonstrated the successful folding of the protein 1D3Z (to within 2.4 Å of the X-ray structure) using only N-H RDCs from one alignment medium.

**Conclusions:**

The additional GUI features of REDCRAFT combined with the NEF compliance have significantly increased the flexibility and usability of this software package. The improvements of the core algorithm have substantially improved the robustness of REDCRAFT in utilizing less experimental data both in quality and quantity.

## Background

Faster and more cost-effective methods of characterizing protein structures are of paramount importance in the development of personalized medicine. While there have been substantial developments in reducing the cost, and increasing the speed of sequencing genomic data [1–4], there has been relatively little advances in improving the characterization of protein structures [5]. In addition to the existing disparity in genetic versus proteomic information, the vast majority of the characterized protein structures belong to a very specific and limited category of proteins. For instance, while it has been estimated that 30% of the human proteome consists of membrane proteins, this important class of proteins is represented by approximately 120 proteins in current databases [6, 7]. Such observed disparities are rooted in the lack of new approaches to structure calculation that overcomes the existing barriers in structural determination of proteins [8, 9].

In recent years, the use of Residual Dipolar Coupling (RDC) data acquired from Nuclear Magnetic Resonance (NMR) spectroscopy has become a potential avenue for a significant reduction in the cost of structure determination of proteins [7]. In addition, RDC data have been demonstrated to overcome some long-standing challenges in NMR spectroscopy such as structure determination of membrane proteins [10–14], recognition of fold families [15] and the concurrent study of structure and dynamics of proteins [16–24]. Recent work [25–30] has demonstrated the challenges in structure calculation of proteins from RDC data alone, and some potential solutions have been introduced [26, 27, 30–33]. One such approach named REDCRAFT [11, 21, 25] has been demonstrated to be successful in structure calculation of proteins from a reduced set of RDC data (and therefore reduced cost). While REDCRAFT has been very successful compared to other approaches, it exhibits some limitations that result in reduced usability and flexibility. In this work, we present usability and methodology `improvements to REDCRAFT that aim to address these limitations. To increase the usability, we have incorporated a powerful Graphical User Interface (GUI), integrated it with molecular visualization software, and adopted the newly approved NMR Exchange Format [34] (NEF), to name a few. REDCRAFT’s core methodology has been revised to allow calculation of protein structures under challenging conditions. More specifically, we improve the decimation routine as well as incorporate new dihedral restraints mined from the PDBMine [35] database. To evaluate the updates, we present and discuss structure calculation of proteins using novel sets of RDC data that REDCRAFT, under lower signal to noise conditions as well as with sparse sets of RDCs. The REDCRAFT package is purely developed in C++ according to valid software development principles and is freely available for download via Bitbucket repository (https://bitbucket.org/hvalafar/redcraft/).

### Residual dipolar couplings

RDCs can be acquired via NMR spectroscopy and the theoretical basis of their interaction had been established and experimentally observed in 1963 [36, 37]. RDC data has become a more prevalent source of data for structure determination of biological macromolecules in recent years due to the availability of alignment media [38] and substantial improvements in NMR instruments. Upon the reintroduction of order to an isotropically tumbling molecule, RDCs can be easily acquired. The alignment medium can impose restricted tumbling through steric, electrostatic, or magnetic interaction with the protein. The RDC interaction between two magnetically active nuclei can be formulated as shown in Eq. (1).

1 $$ {D}_{ij}={D}_{max}\left\langle \frac{3{\mathit{\cos}}^2\left({\theta}_{ij}(t)\right)-1}{2}\right\rangle $$

2 $$ {D}_{max}=\frac{-{\mu}_0{\gamma}_i{\gamma}_jh}{{\left(2\pi r\right)}^3} $$

In this equation, *D*_*ij*_ denotes the residual dipolar coupling in units of hertz between nuclei *i* and *j.* The *θ*_*ij*_ represents the time-dependent angle of the internuclear vector between nuclei *i* and *j* with respect to the external magnetic field of the NMR instrument, and the angle brackets signify time averaging. In Eq. (1), *D*_*max*_ represents a scalar multiplier dependent on the physical properties of the two interacting nuclei and is further described in Eq. (2). In this equation, *γ*_*i*_ and *γ*_*j*_ are nuclear gyromagnetic ratios of nuclei *i* and *j* respectively, *r* is the internuclear distance (assumed fixed for directly bonded atoms), *h* is the modified Planck’s constant, and μ_0_ is the permeability of free space. Additional description and alternate formulations of eqs. 1 and 2 can be found in the following work [25, 39, 40].

### REDCRAFT structural fitness calculation

While generating a protein structure from a given set of residual dipolar couplings is nontrivial, it is straightforward to determine how well a given structure fits a set of RDCs. REDCRAFT’s core approach utilizes this principle in order to produce a viable protein structure. Through algebraic manipulation of Eq. (1) RDC interaction can be represented as shown in Eq. (3),

3 $$ {D}_{ij}={v}_{ij}\ast S\ast {v}_{ij}^T $$

where *S* represents the Saupe order tensor matrix [9] and *v*_*ij*_ denotes the normalized interacting vector between the two interacting nuclei *i* and *j*. REDCRAFT takes advantage of this principle by quantifying the fitness of a protein to a given set of RDCs (in units of hertz) and calculating a root-mean-squared deviation as shown in Eq. (4). In this equation *D*_*ij*_ and *D’*_*ij*_ denote the computed and experimentally acquired RDCs respectively, *N*, represents the total number of RDCs for the entire protein, and *M* represents the total number of alignment media in which RDC data have been acquired. In this case, a smaller fitness value indicates a better structure.

4 $$ Fitness=\sqrt{\frac{\sum_{j=1}^M\ {\sum}_{i=1}^N\ {\left({D}_{ij}-D{\prime}_{ij}\right)}^2}{M\times N}} $$

The REDCRAFT algorithm and its success in protein structure elucidation have been previously described and documented in detail [11, 25]. Here we present a brief overview. REDCRAFT calculates structures from RDCs using two separate stages. In the first stage (*Stage-I*), a list of all possible discretized torsion angles is created for each pair of adjoining peptide planes. This list is then filtered based on allowable regions within the Ramachandran space [36]⁠. The list of torsion angles that remain is then ranked based on fitness to the RDC data. These lists of potential angle configurations are used to reduce the search space for the second stage.

*Stage-II* begins by constructing the first two peptide planes of the protein. Every possible combination of angles from *Stage-I* between peptide planes *i* and *i + 1* are evaluated for fitness with respect to the collected data, and the best *n* candidate structures are selected, where *n* denotes the search depth. The list of dihedral angles corresponding to the top *n* structures is then combined with every possible set of dihedral angles connecting the next peptide plane to the current fragment. Each of these candidate structures is evaluated for fitness and the best *n* are again selected and carried forward for additional rounds of elongation. All combination of dihedral angles worse than the best *n* are eliminated, thus removing an exponential number of candidate structures from the search space. This elongation process is repeated iteratively, incrementally adding peptide planes until the entire protein is constructed.

## Implementation

### Usability updates to the REDCRAFT software package

Several changes have been made to the REDCRAFT package to increase usability including reorganization, documentation, addition of a graphical user interface as well as adoption of NEF standards. These developments are outlined in the following subsections.

#### Reorganization, documentation and addition of GUI

The initial version of the REDCRAFT software package was only accessible through a Linux command line environment. Several changes have been incorporated to allow REDCRAFT to be mostly platform-agnostic, and it is now able to be compiled and executed on any Linux, BSD, or Unix system, including MacOS. Dependencies have also been updated such the latest version of the GNU C Compiler can be used for compilation. In addition, CMake [41] was integrated to all for dynamically generated makefiles that are suitable for an individual machine.

Regardless of the operating system, the command line environment could be cumbersome to use, especially for novice users. To create a more streamlined analysis pipeline, the project was reorganized to allow all REDCRAFT binaries and scripts to run from a single command instead of scattered individual pieces, thereby encapsulating the project and facilitating simpler use. This is accomplished by only including a single binary, `redcraft` in the user’s path that acts as command interpreter for the entire REDCRAFT project.

Additionally, a documentation system was put in place (http://redcraft.readthedocs.io/) that allows new documentation to be built and updated upon every update to REDCRAFT. This documentation details the steps necessary to compile the entire REDCRAFT suite, as well as dependencies. The documentation may be easily exported as HTML, DOCX, or PDF document formats for offline reference.

Finally, a modern Qt5 GUI system was developed to facilitate the usage of REDCRAFT even further. The GUI, written in C++ with Qt5, is fast and available uniformly across all platforms. The GUI contains tools to run *Stage-I* and *Stage-II*, reads config files, and allows for preliminary analysis of output files. Invocation of the GUI is performed by running either `redcraft gui` or `redcraft gui [path]` (to immediately launch the GUI in that directory).

#### Adherence to NEF standards

**Fig. 1:**
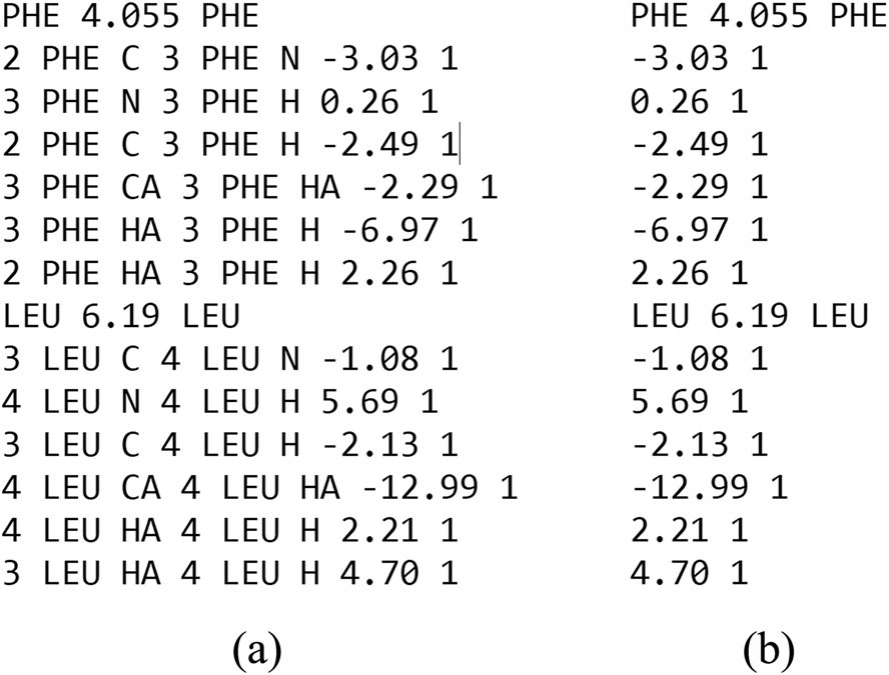
An example of equivalent (**a**) NEF RDC file and (**b**) legacy REDCRAFT RDC file

The previous version of REDCRAFT utilized a rigid file format by allowing the analysis of only six specific RDC vectors (per residue) and their corresponding error values (example shown in Fig. 1b). These six RDC classes represented the most prevalently collected vectors in the field of NMR at the time of REDCRAFT’s creation. Since then, due to advances in instrumentation, introduction of new alignment media, and data acquisition techniques, a much wider range of RDCs can be collected to aid in structure calculation. To address issues such as this the NMR community introduced the NMR Exchange Format [34] (NEF). NEF is a standard for the representation of all NMR restraints and accompanying data. NEF was created from a series of workshops and consultations with developers of NMR structure determination software developers to streamline the pipeline of structure determination programs. The NEF formulation of RDCs is much more flexible in its definitions (an example is shown in Fig. 1a). NEF lists the name, residue number, and residue name of both atoms associated with each RDC along with the RDC value and uncertainty. To accommodate the robust possibilities of RDC values that NEF could contain, REDCRAFT’s computational engine was expanded to handle any combination of the interacting nuclei along the backbone of a protein. The introduction of this standard has allowed the structure determination of proteins with data that was not possible before. To remain backward compatible, a conversion script is available that will convert the legacy format into the NEF format. This conversion script has also been integrated into the GUI.

### Methodology updates to the REDCRAFT software package

#### Improvements of decimation methodology

REDCRAFT’s core principle approach is to generate plausible structures in a combinatorial fashion and evaluate their fitness to the experimental data. To address the intractability of combinatorial approaches, REDCRAFT has incorporated a static-decimation strategy (previously described in [25]) to reduce a large number of quasi-acceptable structures into a smaller and more manageable subset of structures by selecting representative structures. The static-decimation process utilizes user-specified parameters in order to balance the two competing objectives of examining a larger pool of structures versus the computational demands of a larger and more robust search for structures. Proper selection of these parameters is normally a simple process for typical data but becomes impossible for more noisy data. Consideration of structures with poor fitness to the data is unnecessary accommodation under high signal to noise ratio. However, under the conditions of low signal-to-noise ratio, the true structure is more likely to be subjected to early elimination based on poor fitness to the data.

The new version of REDCRAFT overcomes the limitation of the static-decimation process by introducing the more intelligent and adaptive dynamic-decimation process. In the dynamic-decimation process, the search and decimation parameters of REDCRAFT are automatically and dynamically adjusted at each stage of the analysis to reflect the quality, and therefore the computational demands of that stage. To accomplish this, a percentage threshold of tolerance (*n*) is set instead of a static user-defined threshold. At each step in the elongation process of the algorithm, only structures with an RDC fitness score less than the current score of the fragment +*n*% will be considered in the decimation pool. Using this new approach, two common and limiting impediments will be corrected. The first is in situations where there is low data density. In areas of low data density, the contribution from the static-decimation routine causes the solution space to grow exponentially, which is manifested in exponentially increasing computational resources (CPU and memory). For example, during the first few steps of elongation there are typically a few RDCs, which result in underdetermined definition of the problem. In such instances a globally defined acceptance criterion would likely include nearly all the possible structural solutions, as all potential structures will have a low RDC fitness score. Dynamic decimation controls this intractable growth rate by only considering structures within *n*% of the current score of the protein fragment. The second scenario appears in areas of highly noisy data. In these areas, contribution from decimation can drop to zero because of poor local structural fitness to the low-quality data. In this scenario, dynamic decimation assures controlled contribution from decimation.

Using the dynamic-decimation process we have investigated the low signal-to-noise instances of structure determinations that were not possible before. For this evaluation we have used the target protein 1A1Z, for which structure calculation has not been successfully completed using RDC data with low signal-to-noise ratios. In our experiments we have pushed the limits of the structure determination of this protein with as much as ±4 hertz of added uniform noise.

#### Incorporation of data-driven dihedral restraints

The protein databank [42] (PDB) currently houses close to 150,000 protein structures. However vast this collection, the data storage format does not allow for easy mining of low-level information such as dihedral angles restraints. However, recently a minable version of the PDB has been created called PDBMine. Using PDBMine, a protein sequence and a rolling window size is inputted. The protein is then fragmented into k-mers using the rolling window. The dihedral angles are then extracted from these fragments and aggregated for a given amino acid and the most likely dihedral is predicted. The resulting information can then be used to generate the candidate angle files created in *Stage-I* of REDCRAFT by varying the predicted angles ± *n* degrees (*n* = 25 in this work). In this work, datasets as low as one RDC per residue in only one alignment medium will be used to characterize ubiquitin. Ubiquitin (1D3Z) was chosen due to the availability of both high resolution RDC data as well as both x-ray and NMR structure to compare results. It has also been the subject of past RDC studies [18, 26, 28, 43–45] that serve as comparisons for the results of this study. To date, there has been no successful attempt of structure characterization with this sparse of data.

### Evaluation protocol

Throughout the process of evaluating the new features of REDCRAFT we have utilized two target proteins 1A1Z and 1D3Z. These two proteins have been selected because they represent helical proteins, appropriate in size for study by NMR spectroscopy, and have been the subjects of previous studies by RDC data. Each of these proteins provide challenging cases. For example, 1A1Z is a difficult protein to characterize due to its helical nature [46] and structural anomalies that force it to sample atypical Ramachandran Space [47]. The protein 1D3Z also provide other unique challenges due to its helical nature and hypothesized internal dynamics. The helical proteins are generally more challenging to study by RDCs since the backbone N-H vectors are in nearly parallel configuration. The dynamical nature of 1D3Z protein will provide a challenging case of establishing its backbone dihedrals. Other additional challenging attributes of each protein that qualifies them for our studies are described in individual sections.

Our evaluation of REDCRAFT’s improved decimation routine proceeded in three main steps. During the first step, the known structure of 1A1Z was used to generate simulated RDC data using typical order tensors previously used in several studies [25, 48] and the software package REDCAT [49]. The RDC set simulated included the four of the previously available RDC vectors as well as two new vectors ([H^⍺^-C^⍺^, N-C^⍺^]) that were previously unusable in REDCRAFT. Evaluation of a new methodology such as REDCRAFT based on simulated RDC data is of critical value. The use of simulated data allows for exact control over the quality of data, quantification of the performance as a function of signal-to-noise ratio, and proper assessment of time and space complexity of an algorithm as a function of data quality, to name a few.

In addition, to test the utility of incorporating data driven dihedral angles, the protein 1D3Z, the NMR structure of ubiquitin, was used. Due to the availability of experimental RDCs for 1D3Z, no additional synthetic RDCs were generated. Previous results for 1D3Z using REDCRAFT have shown that for high resolution structure calculation, at least two RDC vectors in two alignment media are required. To test the new dihedral restraints, we will attempt to decrease the total RDCs needed.

During the second phase of evaluation, the simulated RDC data are utilized by REDCRAFT to generate a protein structure. During this phase of the experiment, the REDCRAFT’s RDC-fitness score was used to evaluate the success of REDCRAFT. If successful, the viable structures should exhibit an RDC-fitness to the data that is in the same order of the experimental error (related to the signal-to-noise).

Finally, during the third step, the computed structure is compared to the starting structure (the ground-truth) in order to ascertain the success of REDCRAFT. To evaluate structural similarity, the bb-rmsd (backbone root mean squared deviation) between resulting REDCRAFT structures and the target structure was calculated. The measure of bb-rmsd is prevalently used to establish the structural similarity between two proteins. Values under 3.5 Å can signify the success of REDCRAFT under noisy data conditions, while values under 2 Å can be interpreted as strong evidence for structural similarity.

## Results/discussion

### Integration of graphical user Interface

The Graphical User Interface (GUI), written in Qt5, was integrated seamlessly into the REDCRAFT package utilizing CMake. Qt5 contains CMake bindings to link all the necessary Qt dependencies, therefore the end user will notice no difference between compiling the REDCRAFT engine and the GUI itself. The GUI can be launched directly from the command line so that it may immediately open the current working directory, or it may be launched from its binary. REDCRAFT and subsequently REDCRAFT GUI runs seamlessly on all flavors of Linux as well as macOS. Dependencies for this version of REDCRAFT are the GCC G++ Compiler, OpenMP (used for parallelization of processing), Qt5 with Charts (for GUI support), and Python 3 and Perl (for auxiliary script support). Instructions for installation of all dependencies can be found in the REDCRAFT documentation (https://redcraft.readthedocs.io/).

**Fig. 2:**
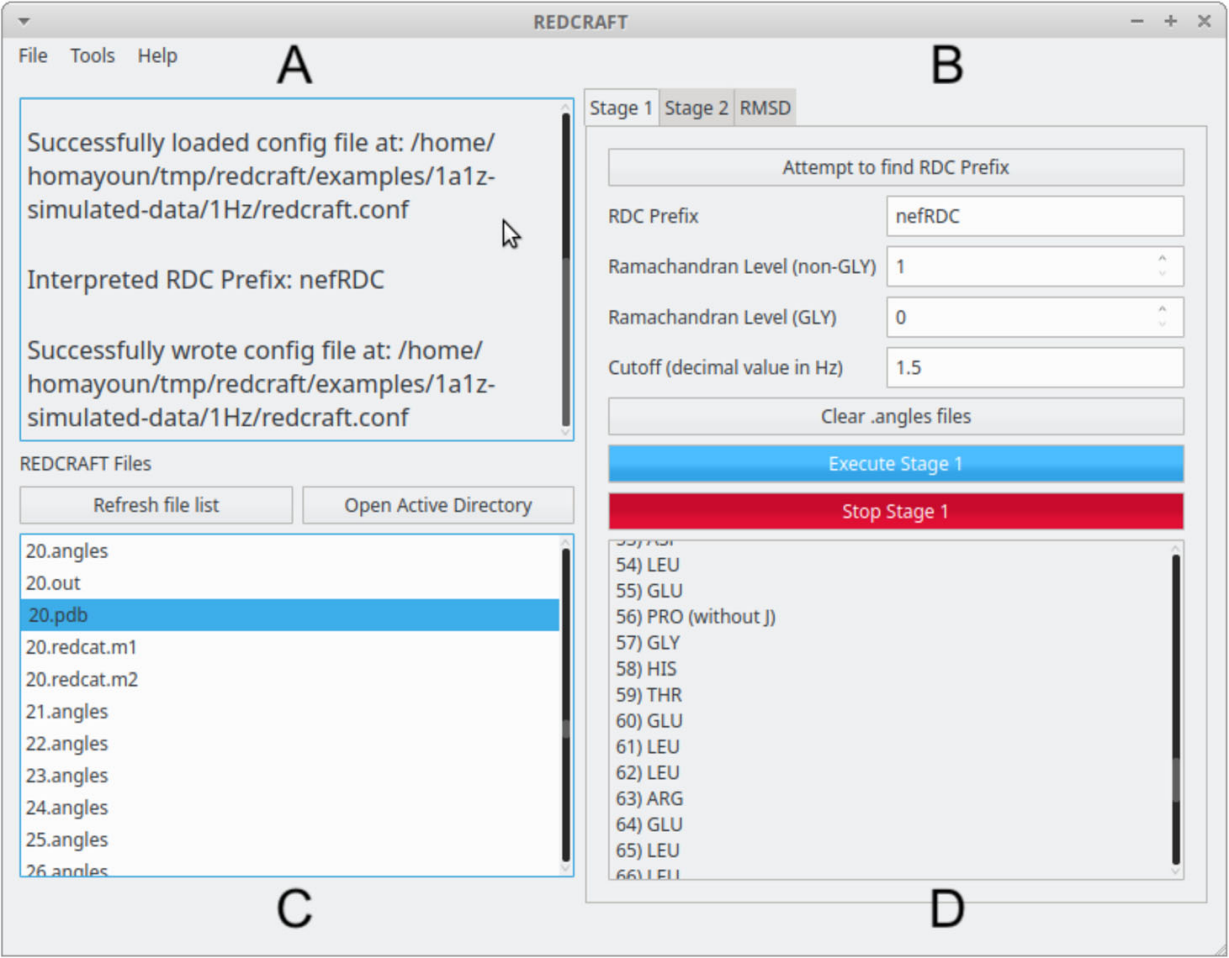
The main REDCRAFT GUI implemented in Qt5

**Fig. 3:**
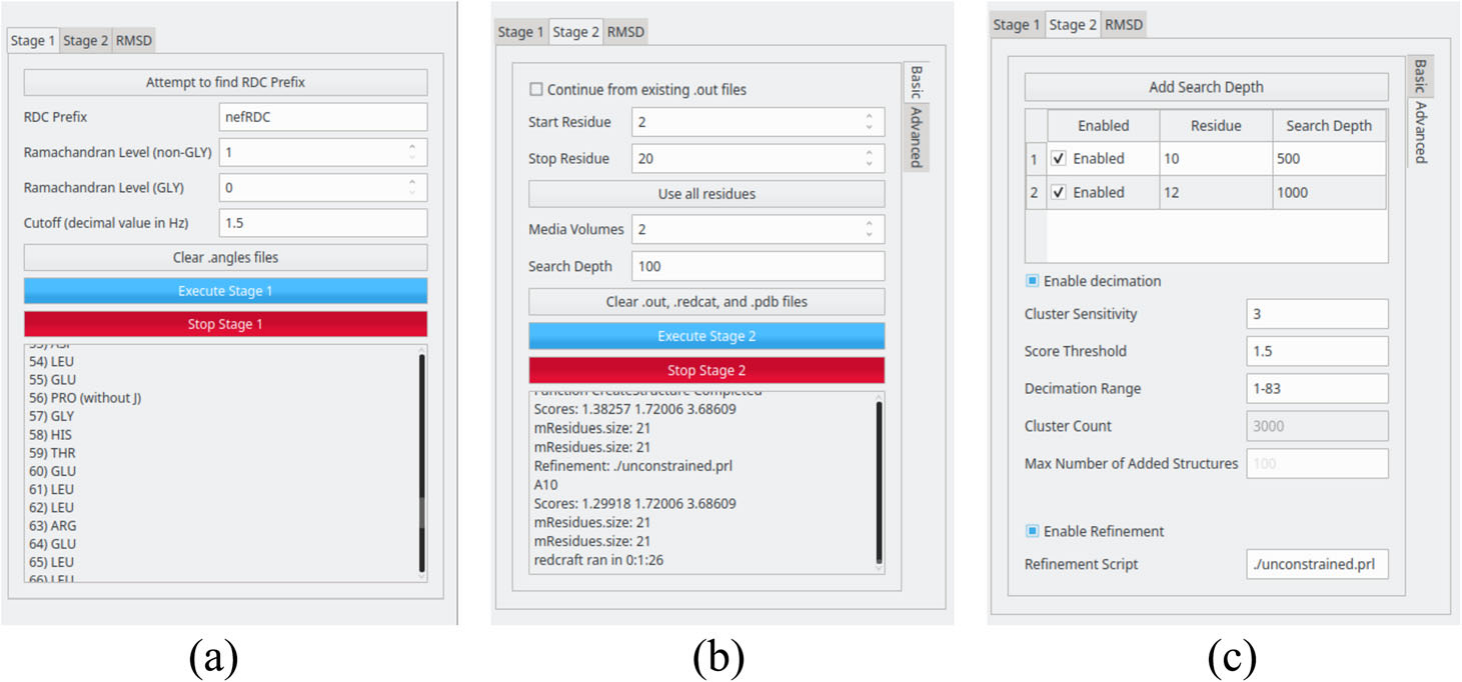
Three examples of dialogues that can be triggered by REDCRAFT at various stages of its analysis

After executing the GUI, the user will be presented with the screen shown in Fig. 2. The initial screen consists of four panels. The first panel (Panel A) displays a greeting message as well as some “quick tips” to aid the user in utilization. Panel B loads the run parameters for *Stage-I* and *Stage-II*. Tabs allow for easy navigation between the two stages. Panel C shows all files present in the user’s working directory, that is, the folder in which the REDCRAFT GUI was started in. This working directory can be changed via File- > Open Directory at the top left of the GUI. In Panel D the output of each stage of execution is printed. For instance, if the “Execute Stage 1” button is pressed then the results of Stage-I angle creation will be shown (see Fig. 3a and b as examples). When in the “Stage 2” tab of Panel B, if the “Execute Stage 2” button is pressed then the results of Stage-II calculation will be shown in Panel D. When the “Advanced” tab is selected in the Stage 2 tab on Panel B, the panel expands to fill the entire column (as seen in Fig. 3c) and additional parameters are shown. At any time during the execution of either stage, the process can be stopped by pressing the stage’s respective “Stop” button (shown in red on Panel B).

After executing the REDCRAFT analysis through its GUI, the resulting config file follows the standard INI format, but with comment support. The user is free to modify the configuration file directly, but the GUI will automatically eliminate any additional user comments in order to maintain backward compatibility.

### Results of structure calculation using improved decimation method

**Fig. 4:**
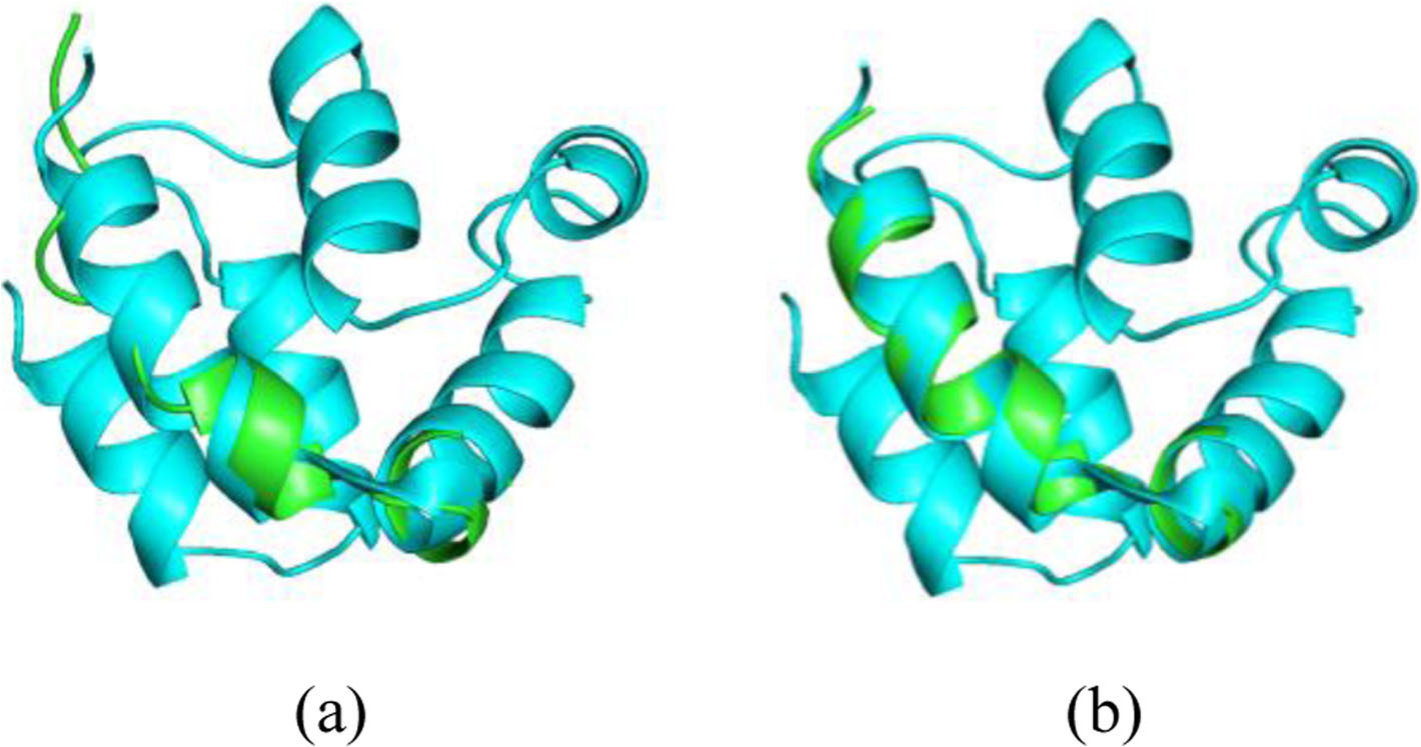
Computed structure of 1A1Z with 4 Hz of experimental error (**a**) produced by the legacy version, and (**b**) by the improved decimation procedure

**Fig. 5:**
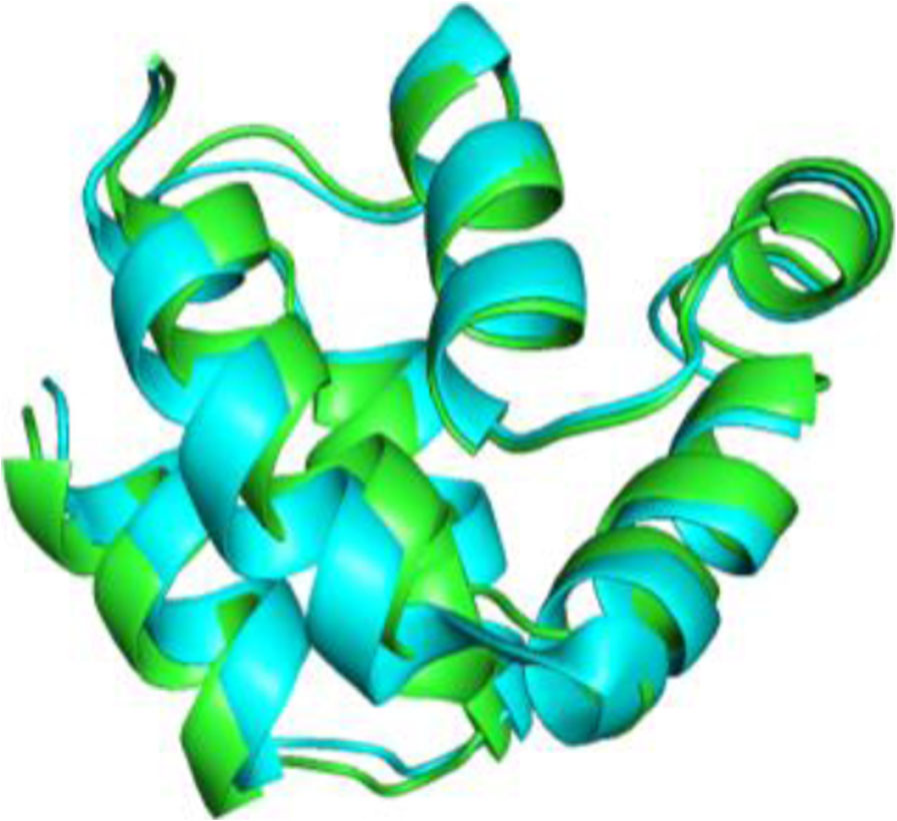
A comparison of the structural similarity between the X-ray structure of 1A1Z and the computed structure of the entire structure by REDCRAFT using new RDC vectors and the NEF format

The new version of decimation is universally faster than the previous version. Figure 4 shows the results of the first 20 residues of 1A1Z (using RDC data with ±4 Hz of error) folded with the previous version of decimation compared to the same segment folded using the new decimation method using identical search parameters. The 20-residue (out of 83 total) segment of 1A1Z was selected due to the excessive space requirement of the previous version of decimation. The previous version required 4 h of analysis time, at the end of which the final structure exhibited a bb-rmsd of 1.589 Å to the reference structure (RDC fitness score of 2.21, results shown in Fig. 4a). However, the extension of this fragment required memory in excess of the 16GB of the host computer and therefore did not complete the full analysis of the protein within a week. The new version of the decimation completed this exact segment on the same host computer in about 4 min and produced a structure with backbone bb-rmsd similarity of 0.946 Å to the reference structure (RDC fitness score of 2.19, shown in Fig. 4b). Of the greater importance is the success of the new version of REDCRAFT in providing a full structure of 1A1Z (illustrated in Fig. 5 and discussed in the next section) that was never completed by the previous version of the software.

### Results reconstruction of proteins using NEF format

The changes to the core REDCRAFT engine to accept NEF format enable it to perform the structure calculation of proteins based on a flexible set of RDC data. RDC pairs that were unavailable in the old version are now able to be used for reconstruction. For example, 1A1Z with [H^⍺^-C^⍺^, N-C^⍺^] RDC data in two alignment media with 0 Hz of simulated noise can now be folded with REDCRAFT. Using the new decimation approach, REDCRAFT produced the final structure of 1A1Z with a bb-rmsd of 1.404 Å and an RDC fitness score of 0.835 when compared to X-ray structure of 1A1Z (Fig. 5). This is a substantial achievement in the successful folding of a protein with flexibly defined RDCs.

However, it should be noted that this modification causes a slight increase in runtime that can vary from 1 to 5% slower than the previous version. The time requirements were benchmarked by performing structure calculation of the same protein, using the same set of RDCs in both the previous and NEF-compatible version (results shown in Fig. 5). Typically, the new version of REDCRAFT completes within a minute of the previous version for an analysis that takes approximately 45 min, and therefore the slower performance is considered negligible.

### Incorporation of data-driven dihedral restraints

The protein sequence for ubiquitin (76 residues) was submitted to PDBMine with a rolling window size of six. The resulting dihedral predictions for each amino acid was then used to create dihedral restraints by varying them +/− 25 degrees in steps of 5 degrees to be used in *Stage-I* of REDCRAFT. The structure was then calculated with a varying set of RDC data both with and without the PDBMine-based dihedral restraints. For each set of data, a figure depicting the alignment was produced, in which the target structure is shown in green, the structure determined without the dihedral restraints in magenta and the structure determined using the dihedral restraints in blue.

**Fig. 6:**
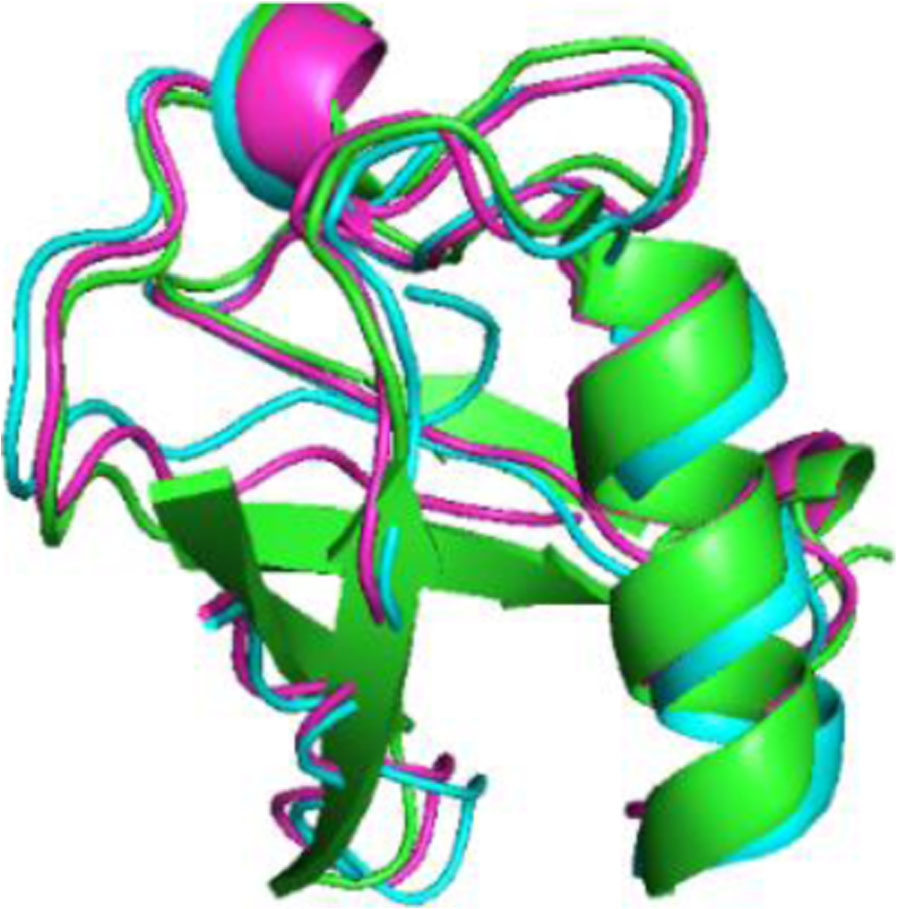
Alignment for the structure with dihedral restraints (magenta), without (blue) and x-ray structure of ubiquitin (green) for the first set of RDCs ([C′’-H, N-H]× x2)

The first set of data (results shown in Fig. 6) included [C′-H, N-H] from two alignment media. The resulting structure without the use of the dihedral restraints was 2.8 Å from the x-ray structure whereas using the dihedral restraints resulted in a structure that was just 1.4 Å away from the target.

**Fig. 7:**
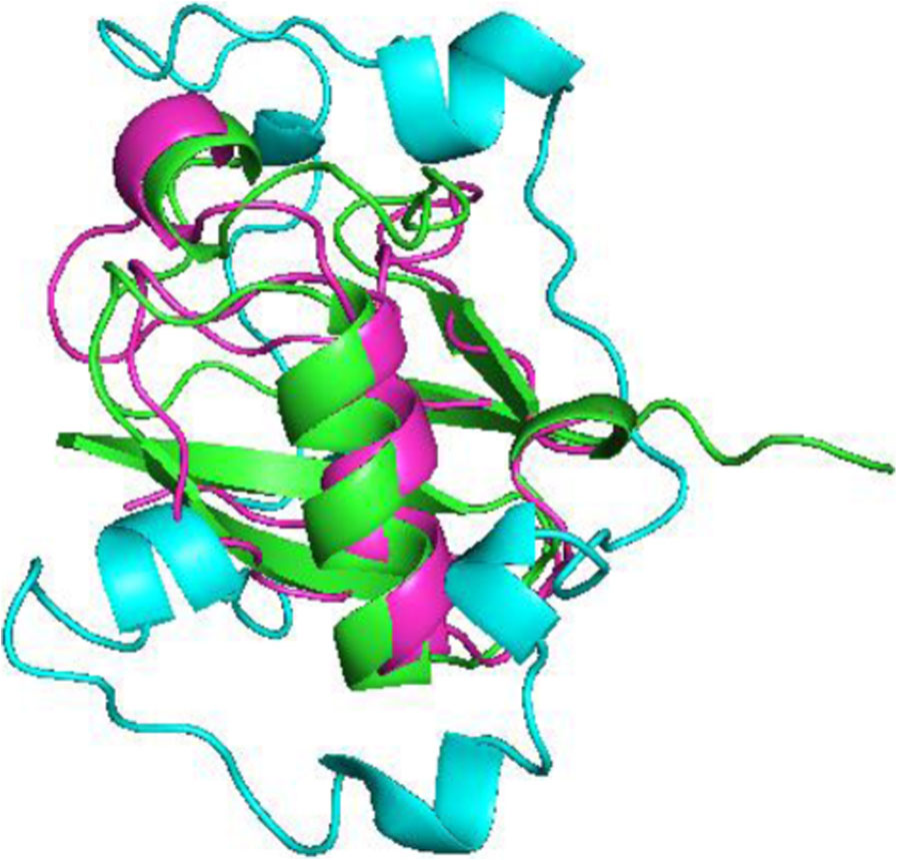
Alignment for the structure with dihedral restraints (magenta), without (blue) and x-ray structure of ubiquitin (green) for the second set of RDCs (N-Hx2)

The second set of data (results shown in Fig. 7) included only [N-H] RDCs in two alignment media. The resulting structure without the dihedral restraints exhibited a bb-rmsd of 11.6 Å whereas the structure that utilized dihedral restraints exhibited structural deviation of just 2.0 Å.

**Fig. 8:**
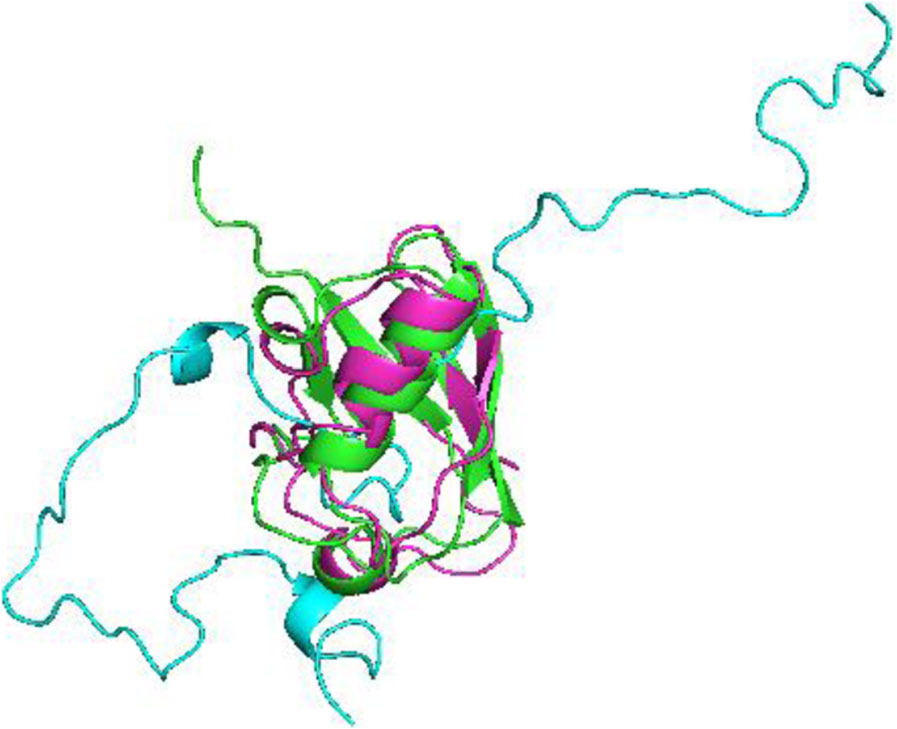
Alignment for the structure with dihedral restraints (magenta), without (blue) and x-ray structure of ubiquitin (green) for the second set of RDCs (N-Hx1)

The last set of data (results shown in Fig. 8) included only [N-H] RDCs from just one alignment medium. The structure without dihedral restraints was over 21.1 Å away from the target structure whereas the structure calculation using dihedral restraints was just 2.4 Å away.

Detailed results are shown in Table 1 for each of the datasets. The bb-rmsds in this table clearly show that the incorporation of data-driven dihedral increases the structure calculation ability of REDCRAFT. In addition, structural alignment of the three proteins from each set were aligned using a multiple structure alignment tool called MSTali [50]. Using the first set of RDCs, the three resulting structures retain 67 residues in common structurally. This indicates high level of structural similarity. However, when the dataset is reduced to the second and third set then the core residues in common drops to 29 and 22 respectively. This reinforces the structural dissimilarity of the structures in which the dihedral angles were used and those in which they were not.

**Table 1:** Results for each of the datasets is shown

Set	RDCs (# Align Media)	BB-rmsd Without Dihedrals	BB-rmsd With Dihedrals
1	[C′-H, N-H] (2)	2.8	1.4
2	[N-H] (2)	11.6	2.0
3	[N-H] (1)	21.1	2.4

### Additional scripts, functionality, and features

During structure calculation, thousands of different phi/psi combinations are explored. Currently, the REDCRAFT algorithm will automatically generate a .pdb file for the top structure as each amino acid is added to the structure. However, one may be interested in considering an ensemble of the top *N* structures, not just the “best” structure. To facilitate this analysis, pdbgen and pdbgen2 have been added which both generate .pdb files based on a string of phi/psi angles and a string of amino acids. Pdbgen can generate structures directly from the .out files that are created during a run of REDCRAFT and is able to read single character residue names. Pdbgen2, which does not require any options and only takes in a string of phi/psi angles and a string of amino acids as its arguments, is simpler to use and desirable for quick pdb construction. The pdbgen collection accommodates both basic and comprehensive structure generation from phi/psi angles. These programs can also function as standalone programs for quick pdb generation and verification where the other features of REDCRAFT are not necessary. The pdbgen tools will eventually make up part of the REDCRAFT GUI analysis suite where they can be better employed to help users find exactly where the intermediate protein structure may deviate during structure generation.

### Conclusions/future work

In this work, we have presented significant improvements to the REDCRAFT software package in the important areas of usability, accessibility, and core methodology. The inclusion of a GUI makes the software more usable by a wider audience. Incorporation of NEF standards makes the software compliant with a large suite of other widely available NMR software packages. In addition, the NEF import file allows for increased flexibility of RDCs that can be utilized by REDCRAFT which will allow structure calculations of more complex and larger proteins, such as those that have been perdeuterated due to size. We have also shown that the improved decimation method allows the method to be used to calculate proteins that it was unable to complete before due to experimental noise. In addition, we presented incorporation of a dihedral restraint that was mined from the PDBMine database. Using these restraints, the structure of ubiquitin was characterized using just one RDC from one alignment medium. Structure calculation with so few RDCs per residue has, to date, never been achieved. Lastly, we introduced new standalone functionality to produce .pdb files from only phi/psi angles which is useful when analyzing ensembles of structures.

In future work, we plan to extend the REDCRAFT algorithm to also be capable of characterizing nucleic acids.

### Availability and requirements

**Project name:** REDCRAFT v2.

**Project home page:** https://bitbucket.org/hvalafar/redcraft/

**Operating system(s):** Any Linux, BSD, or Unix system, including MacOS.

**Programming languages:** C++, python, Perl.

**Other requirements:** gcc (version 7.3 or higher), CMake (version 3.10.2 or higher), OpenMP (version 5.0 or higher), Qt (version 5.12 or higher), python (version 3.6.9 or higher) and Perl (version 5.26 or higher).

**License:** GNU GPL.

**Any restrictions to use by non-academics:** None.

## Data Availability

The raw data used in this study can be acquired from the authors upon reasonable request.

## Abbreviations

NMR: Nuclear Magnetic Resonance
NOE: Nuclear Overhauser Effect
RDC: Residual Dipolar Coupling
REDCRAFT: Residual Dipolar Coupling based Residue Assembly and Filter Tool
GUI: Graphical User Interface
NEF: NMR Exchange Format
PDB: Protein DataBank
bb-rmsd: Back-Bone Root Mean Squared Deviation
